# Precious metal clusters as fundamental agents in bioimaging usability

**DOI:** 10.3389/fchem.2023.1296036

**Published:** 2023-11-09

**Authors:** Xiaoxiao He, Shaojun Liu, Xi Hu, Xiongyi Huang, Hehua Zhang, Xiang Mao

**Affiliations:** ^1^ Department of Medical Engineering, Daping Hospital, Army Medical University, Chongqing, China; ^2^ State Key Laboratory of Ultrasound in Medicine and Engineering, College of Biomedical Engineering, Chongqing Medical University, Chongqing, China; ^3^ Chongqing Key Laboratory of Biomedical Engineering, College of Biomedical Engineering, Chongqing Medical University, Chongqing, China

**Keywords:** precious metals, clusters, fluorescent property, optical stability, bio-imaging

## Abstract

Fluorescent nanomaterials (NMs) are widely used in imaging techniques in biomedical research. Especially in bioimaging systems, with the rapid development of imaging nanotechnology, precious metal clusters such as Au, Ag, and Cu NMs have emerged with different functional agents for biomedical applications. Compared with traditional fluorescent molecules, precious metal clusters have the advantages of high optical stability, easy regulation of shape and size, and multifunctionalization. In addition, NMs possess strong photoluminescent properties with good photostability, high release rate, and sub-nanometer size. They could be treated as fundamental agents in bioimaging usability. This review summarizes the recent advances in bioimaging utilization, it conveys that metal clusters refer to Au, Ag, and Cu fluorescent clusters and could provide a generalized overview of their full applications. It includes optical property measurement, precious metal clusters in bioimaging systems, and a rare earth element-doped heterogeneous structure illustrated in biomedical imaging with specific examples, that provide new and innovative ideas for fluorescent NMs in the field of bioimaging usability.

## Introduction

Bioimaging technology is an important research tool to view biological functions in real-time and to elucidate various physiological functions of organisms (Karlas et al., 2021; Mazumder et al., 2022; Ju et al., 2023), this approach has minimal interference with life processes and allows the direct acquisition of microstructural images by using optical or electron microscopy (Yang et al., 2022; Sobhanan et al., 2023). The analysis of the resulting images is used to understand different physiological processes in biological cells. In addition, bioimaging is widely used to acquire data on the three-dimensional structure of an observed object without physical interaction (Klymchenko et al., 2021). Biological imaging covers a wide range of modalities, including X-ray, ultrasound, computed tomography (CT), positron emission computed tomography (PET), magnetic resonance imaging (MRI), etc (Wen et al., 2020). Among them, fluorescence imaging technology plays an important role in whole imaging medical works. Its advantages are divided into such categories as high sensitivity, easy observation, and simple instrumentation, which mainly utilize the change of fluorescence characteristics to obtain optical images (Collot, 2021). Also, it is necessary to introduce exogenous fluorescent materials as a contrast agent to calibrate specific cells, tissues, and organs (An et al., 2020; Zhang et al., 2020; Wang et al., 2021). Due to the lack of endogenous fluorescent materials in many biological structures and processes, it is difficult to utilize the intrinsic fluorescence of biological samples for imaging characterizations. Therefore, the potential of fluorescent agents is recognized as a new type of typical functional material, that could be utilized in this area. For example, in the field of bioimaging, they have the advantages of excellent fluorescence performance, tunability, multifunctionality, and high photostability (Cai et al., 2021; Guo et al., 2021). Fluorescene imaging technology can provide comprehensive detection methods from cells, isolated tissues, and living biological samples in terms of structural and dynamic information (Drozdov et al., 2022; Song et al., 2022) in the interdisciplinary fields of materials, optics, and biomedicines. Among them, noble metal nanoclusters, as a new type of fluorescent agent recently developed, have a broad development prospect in the field of bioimaging due to their unique optical properties, biocompatibility, high contrast, tunability, and targeting. In this review, we mainly summarize and outline the properties of precious metal clusters as fluorescent NMs in the context of recent trends and their application in bioimaging usability. Here, there are three different parts fully illustrated: 1) pure-phased metal clusters as fluorescent agents in optical measurements; 2) copper (Cu), silver (Ag), and gold (Au)- based clusters were utilized in bioimaging usability; 3) rare earth element-doped fluorescent materials in imaging applications. Based on the applied research on the functional materials of noble metal clusters, they will gradually come to be widely used in imaging analysis and therapies. Hence, based on the application research of precious metal clusters’ functional materials, it will graduallycome to have extensive application in imaging analysis and treatments.

### Precious metal clusters as fluorescent agents in optical measurement

**FIGURE 1:**
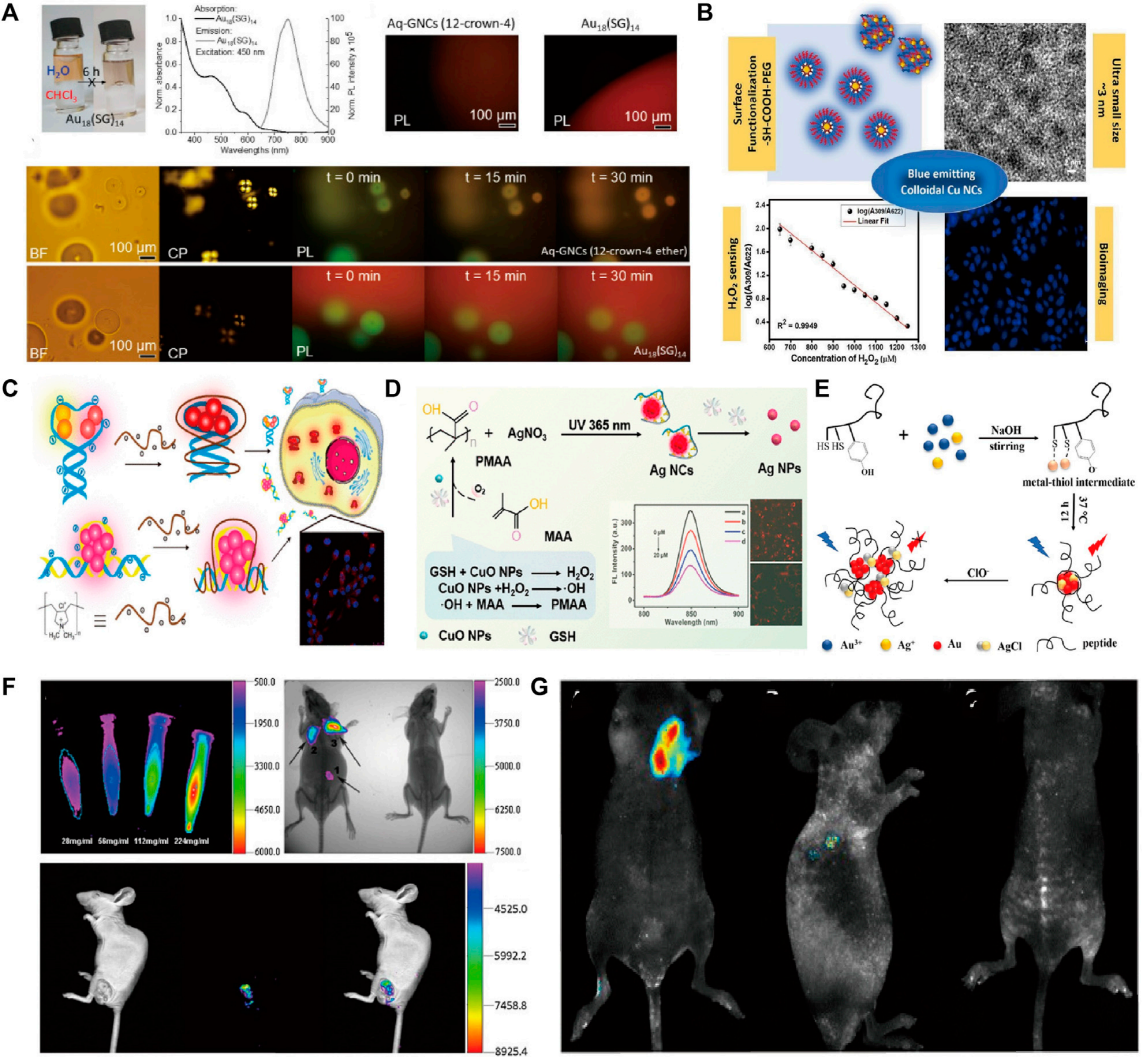
**(A)** Absorption and emission spectra and fluorescence microscopy images of Au_18_GNC in water; **(B)** PEGylated Cu nanoclusters: A nontoxic, multifunctional colloidal system for bioimaging and peroxide sensing; **(C)** The formation of FL DNA-Ag NC/cationic polyelectrolyte complexes for cell imaging; **(D)** Schem of self-Cascade Nanoenzyme of Cupric Oxide Nanoparticles (CuO NPs) Induced *in Situ* Catalysis Formation of Polyelectrolyte as Template for the Synthesis of Near-Infrared Fluorescent Silver Nanoclusters; **(E)** Schematic of synthesis of peptide@Ag/Au NCs and reaction mechanism of peptide@Ag/Au NCs with ClO^−^; **(F)** AuNCs@SiO_2_-FA nanoprobes for fluorescence imaging; **(G)** Representative xenograft tumor nude mice models of Cervical carcinoma *in vivo* imaging. Reproduced from Obstarczyk et al. (2023) with permission of ACS Omega. Reproduced from Chandran et al. (2023) with permission of Biochim Biophys Acta Gen Subj. Reproduced from Lyu et al. (2019) with permission of Anal Chem. Reproduced from Xu et al. (2022) with permission of Analytical Chemistry. Reproduced from Jia et al. (2020) with permission of Talanta. Reproduced from Zhou et al. (2013) with permission of J Nanobiotechnology. Reproduced from Ge et al. (2015) with permission of J Nanobiotechnology.

Fluorescent NMs are of interest for their multifunctional applications in solar cells, biomarkers, imaging, etc. Among them, metallic NMs exhibit strong fluorescence emission and offer great potential for the development of biomarkers and imaging. In particular, precious metal nanoparticles (NPs), which are represented by gold (Au), silver (Ag), and copper (Cu), with unique surface plasmon resonance (SPR) in the range of light from the ultraviolet (UV) to the near-infrared (NIR) (Chakraborty and Pradeep, 2017), and distinctive optical properties such as molecular absorption and strong luminescence, have attracted much attention (Babu Busi et al., 2022). Metal nanoclusters have improved luminescence efficiency and enhanced biocompatibility compared to metal nanoparticles and can escape the renal barrier. Among them, the fluorescent metal clusters exhibit tunable fluorescence from the visible to the near-infrared region. This tunable fluorescence occurs either through molecular-like electronic leaps within the conduction band or due to charge transfer leaps from the ligand to the metal nanoparticles. It was considered a class of fluorescent clusters with novel properties such as ultra-small morphologies, water-solubility, biocompatibility, and so on. By virtue of their usefulness, these precious metal clusters can complement or even replace traditional fluorescent probes in sensor fabrication. This can provide great opportunities for the advancement of imaging technology (Wang et al., 2018), represented by Au nanocluster (NCs), Ag NCs, and Cu NCs. Au NCs have recently been used for bioimaging and other biomedical applications due to their favorable intrinsic optical properties, highly stable chemical properties, and good biocompatibility (Chen et al., 2015). For example, Liu et al. prepared atom-precise Au NCs with 25 gold atoms and 18 peptide ligands, such that the cluster can be used as a NIR-II fluorophore (Liu et al., 2019). Especially in brain imaging, this study found that NIR-II imaging based on Au NCs is able to monitor many small blood vessels and can be used as NIR-II dyes for imaging. The preparation of cluster structures can be realized by using organic ligands in order to optimize their performance with better biological properties. In a recent research report, Obstarczyk et al. prepared ultrasmall Au NCs using 12-crown-4 ligand capped (Obstarczyk et al., 2023). Such nanoclusters were amphiphilic and could be successfully transferred between aqueous and organic solvents while maintaining their physicochemical integrity. They can be used as probes for light (because they emit near-infrared fluorescence) and electron microscopy (because of the high electron density of Au) in multimodal biological imaging (Figure 1A). Wang et al. used *in situ* self-assembly to biosynthesize fluorescent Au nanocluster-DNA (Au-DNA) complexes for precise bioimaging and safe, targeted cancer therapy (Wang et al., 2019).

### Copper (Cu), silver (Ag), and gold (Au) based clusters utilized in bioimaging systems

The work on photoluminescence characterization by utilizing Cu clusters in biomedical imaging works. Cu materials were thought to be one functional substrate material due to their abundance and relatively low cost (Lai et al., 2020; Akhuli et al., 2021; Saraf et al., 2021) in nature. In addition, as one typical application of biosensors, it was modified by different surfactants to form functional agents. As in bioimaging works, Chandran et al. (2023) developed blue-emitting colloids, Cu NCs, using different functional groups (-SH and -COOH). Some characteristics (size, cytotoxicity, and emission properties of Cu NCs) were controlled due to surface functionalization. It protected the particle surface from aggregation and oxidation, mainly through the binding of polymer molecules with thiol and carboxyl groups. The bright blue fluorescence emitted by HeLa cells treated with acetic acid (sample code: CAGP) (Figure 1B) showed excellent bioimaging properties. The functional group in molecules was used as a simple and cost-effective method to synthesize glutathione-coated copper nanoclusters (Cu-GSH NCs) with strong, bright red fluorescence (625 nm) (Chandran et al., 2020). It was used as an effective pH-based bio-imaging probe for the detection of cancerous cells and had the potential to be used for label-free subcellular organelles for tracking and labeling. Also, Ag clusters received tremendous attention for biomedical applications due to their low toxicity, size-dependent, and emission properties (Liu et al., 2017). Despite the relatively low photo-oxidative stability of Ag, it has been shown that very strong fluorescence signals can also be generated by using different ligands in the formatting process (Tao et al., 2015; Song et al., 2016). In a recent study, Lyu et al. (2019) utilized cationic polyelectrolytes to modify fluorescent DNA-Ag NCs through electrostatic interactions between the positive polymer backbone and the negatively charged phosphate groups of the DNA strand. The experimental results showed a 3-fold enhancement of fluorescence emission from Ag NCs for rapid cellular imaging, increased stability in the internal environment, and enhanced cellular uptake of DNA-Ag NCs (Figure 1C). Xu et al. (2022) prepared Ag clusters with near-red fluorescence by using polymethacrylates (PMAA) as a template. Which was used as a glutathione (GSH) sensing and bioimaging probe (Figure 1D). Yu et al. (2021) stabilized the Ag cluster (BSA-Ag) by using bovine serum albumin (BSA) with near-infrared electrochemiluminescence (ECL) properties. It exhibited a strong anodic ECL spectral peak at 904 nm in aqueous media with excellent bioimaging properties. As one acceptable approach, the integration procedure should be one effective path to construct heterogeneous composites because of their superiority over single metals in terms of electronic, optical, and catalytic properties (Tian et al., 2017; Zhai et al., 2017). Jia et al. (2020) developed peptide-capped Au-Ag clusters for lysosome-targeted imaging of hypochlorite with high fluorescence quantum yield (Figure 1E). Zhou et al. (2013) utilized folic acid (FA)-coupled silica-coated Au clusters in forming AuNCs@SiO_2_-FA probes with biocompatible properties and applied them to fluorescence imaging in mice (Figure 1F). Ge et al. (2015) explored a new strategy for the synthesis of fluorescent Au-Ce NCs by doping trivalent cerium into the crystal seed growth process of Au clusters. Its fluorescent property can be used to achieve highly sensitive *in vitro* or *in vivo* bioimaging of tumor targets (Figure 1G). In addition, Yang et al. (2020) developed a therapeutic nanomedicine (AuNCs-Pt) based on nanocarrier gold nanoclusters (AuNCs), which has the dual function of NIR-I/NIR-II imaging and glutathione scavenging ability. The long emission wavelength of AuNCs-Pt allows deep penetration, enabling high-resolution NIR-II tumor imaging and improved visualization of platinum transport in deep tissues. Moreover, alloy structures such as Ag-Pt (Bootharaju et al., 2017) and Au-Cu (Shellaiah et al., 2019; Shan et al., 2022) have a wide range of applications in the field of bio-imaging, where they are characterized by good light stabilization, strong NIR-II absorption, and better imaging depths. In summary precious metal clusters with good biocompatibility, stable optical properties, and high luminescence efficiency have a wide range of development prospects in the field of bioimaging.

## Rare earth element-doped fluorescent materials in imaging systems

Rare earth-doped nanoparticles (RENPs) have become the preferred candidate for NIR-II imaging due to their attractive features such as a narrow emission spectrum, long fluorescence lifetime, and absence of photobleaching (Qu et al., 2020; Gu et al., 2022; Song et al., 2022). Zhang et al. (2021) prepared two RENPs, NaYF_4_:Yb_20_Er_2_@NaYF_4_ and NaYF_4_:Nd_5_@NaYF_4_, and modified them with poly (ethylene glycol) (PEG) to explore simultaneous imaging in NIR-IIb (1,530 nm, under 980 nm laser excitation) and NIR-IIb. It showed that RENP’s NIR-II fluorescence has a highly synergistic imaging capability in versatile biomedical applications with higher temporal and spatial resolution, respectively. Tan et al. (2018) reported a synthesis of long-lived rare-earth-doped fluoride nanoparticles using a different strategy: core/shell and dopant engineering, which showed intense infrared emission in a second biological window with a luminescence lifetime of close to 1 m. Karthickraja et al. (2021) synthesized calcium fluoride (CaF_2_) nanoparticles by doping them with optimal concentrations of Nd^3+^ and Yb^3+^ as sensitizers and activators. It performed *ex vivo* fluorescence imaging experiments on chicken breast tissues of different thicknesses with a maximum theoretical depth of penetration of 14 mm for near-infrared light. There is a process of surface modification in doped materials, lanthanide-doped materials can be carried out to realize luminescence through energy level jumps with high luminescence efficiency (Li et al., 2022). Alternatively, the loading of fluorescent molecules into organic nanomaterial structures *via* covalent bonding or electrostatic/hydrophobic interactions to generate fluorescent organic nanoparticles (FON) can also enable efficient bioimaging. FON is another classical material with bioimaging properties that is mainly composed of natural or synthetic organic polymers. The related advantages involved luminescence, biocompatibility, and a high signal-to-noise ratio (Asahi et al., 2008; Sinha et al., 2022). In imaging usability, FONs generally showed stable photo-induced characterizations (Blasi et al., 2017), but they also conveyed that small molecules contributed to higher fluorophore density and spatial resolution in whole fabrications (Vargas-Nadal et al., 2022).

## Conclusion and outlooks

This mini-review, mainly describes the recent advancements in fluorescent precious metal clusters, which are used in biomedical imaging applications such as fluorescence imaging and near-infrared region applications. Based on recent research, precious metal clusters could be considered an acceptable unit for fabricating multifunctional constructions. But also, its particular morphology is a fundamental agent in further designing optical characterization modifications. Using precious metal nanoclusters as the basic building blocks can improve the photostability and durability of bio-imaging and achieve better imaging results. In addition, referring to the heterogeneous structure, precious metals can dope with or be doped with different materials for fabricating imaging substitutes. Meanwhile, their optical properties and biocompatibility are mainly represented by precious metal clusters and hetero composites. Doped with rare-earth elements, they can be used as highly sensitive and selective molecular probes for precise imaging at the cellular and tissue levels, providing more accurate diagnostic and therapeutic tools for clinical medicine. By recognizing the fundamental property, these different precious metal clusters played an important role in the early diagnosis of diseases, individualized therapy, and life-accessible research for further challenges.
